# Neurotransmitter system gene variants as biomarkers for the therapeutic efficacy of rTMS and SSRIs in obsessive-compulsive disorder

**DOI:** 10.3389/fpsyt.2024.1350978

**Published:** 2024-05-22

**Authors:** Lingjun Chu, Yidan Wu, Jiajun Yin, Kai Zhang, Yiwen Zhong, Xiwang Fan, Guoqiang Wang

**Affiliations:** ^1^ Clinical Research Center for Mental Disorders, Shanghai Pudong New Area Mental Health Center, School of Medicine, Tongji University, Shanghai, China; ^2^ Brain Science Basic Laboratory, The Affiliated Wuxi Mental Health Center of Jiangnan University, Wuxi Central Rehabilitation Hospital, Wuxi, Jiangsu, China; ^3^ Department of Psychiatry, Chaohu Hospital of Anhui Medical University, 64 Chaohu North Road, Hefei, Anhui, China

**Keywords:** OCD, COMT, SLC6A4, GRIN2B, repetitive transcranial magnetic stimulation

## Abstract

**Purpose:**

This study aims to examine the potential influence of RS4680 (*COMT*), RS16965628 (*SLC6A4*), and RS1019385 (*GRIN2B*) polymorphisms on the therapeutic response to repetitive transcranial magnetic stimulation (rTMS) and selective serotonin reuptake inhibitors (SSRIs) in individuals with obsessive-compulsive disorder (OCD).

**Patients and methods:**

Thirty-six untreated outpatients diagnosed with OCD were recruited and allocated to active or sham rTMS groups for two weeks. The mean age of the participants was 31.61, with 17 males (47.22%) and 19 females (52.78%). Peripheral blood samples (5 mL) were collected from each participant using ethylenediaminetetraacetic acid (EDTA) vacuum tubes for genotyping purposes, clinical evaluation was taken place at baseline and second week.

**Results:**

The A allele of RS4680, C allele of RS16965628, and GG allele of RS1019385 were identified as potential bio-markers for predicting treatment response to OCD treatments (rTMS & SSRIs).

**Conclusion:**

Those genes may serve as bio-markers for the combined treatment of rTMS and SSRIs in OCD. The finding hold promise for further research and the potential implementation of precision treatment of OCD.

**Clinical trial registration:**

https://www.chictr.org.cn, identifier ChiCTR1900023641.

## Introduction

Obsessive-compulsive disorder (OCD) is a chronic psychiatric disorder, characterized by persistent distressing obsessions and/or compulsions that impair the quality of daily life, associated with severe functional impairment (1–3). Previous research studies have indicated that selective serotonin re-uptake inhibitors (SSRIs) and repetitive transcranial magnetic stimulation (rTMS) are effective treatments for OCD (4–9).

The therapeutic efficacy of SSRIs could be supported by the serotonergic hypothesis (10).

The hypothesis postulates the presence of an aberration, presumably a diminution of function, within the serotonergic system in OCD, or posits the implication of the serotonergic system in some capacity in the pathogenesis of OCD (11–13). Thus, the SSRIs could be effective in treating OCD might because they target on the serotonin system (6, 14). However, it could also be argued that the development of the serotonin hypothesis stemmed from the efficacy of SSRIs in treating OCD, there are still numerous other medicines and treatments that are beneficial for OCD patients. Nonetheless, SSRIs is still one of the effective treatment for OCD (15–17).

RTMS is a comparatively new treatment approach for OCD, which was designed for resistent OCD, and those who cannot afford the huge side effects of medicines (4, 18, 19). As a non-invasive neuromodulation technique, rTMS harnesses rapidly altering electromagnetic fields produced by a coil positioned on the scalp to modulate cortical and subcortical function (5, 20, 21). By adjusting stimulation parameters, rTMS can selectively diminish or enhance cortical excitability in specific regions; frequencies equal to or below 1 Hz typically inhibit activity (referred to as low-frequency rTMS), while frequencies at or above 5 Hz typically stimulate activity (referred to as high-frequency rTMS) (22–24). As several studies suggested, low-frequency rTMS administered over the (pre-) supplementary motor area (pre-SMA/SMA) has been observed to reinstate cortical inhibition in the motor cortex among OCD patients, correlating with amelioration in OCD symptoms (20, 25–27).

However, it has been observed that some patients exhibit inadequate response to these therapies. For instance, studies have shown that SSRIs are ineffective for 40-60% of OCD patients (18, 28). Similarly, some studies did not observe adequate therapeutic effect of rTMS on OCD (29–31), and there is insufficient evaluation of the therapeutic efficacy of rTMS treatment on OCD (20, 32). In light of this, the concept of candidate genes that may impact the therapeutic effect of OCD treatments has been proposed (33), they identified the LL allele of *5-HTTLPR* (L/S) in the candidate gene *SLC6A4* as a potential bio-marker for OCD treatment. Since there may be other bio-markers, attention has been directed towards genes within the neurotransmitter systems, including the serotonin transporter gene *SLC6A4*, the glutamate receptor gene *GRIN2B*, and the gene related to serotonin, dopamine, norepinephrine, and epinephrine (COMT) which have been previously identified as candidate genes for OCD (34–38).

As a gene that has already been identified as a potential candidate for OCD treatment, SLC6A4 holds significant value for further investigation (33). The SLC6A4 gene can encode the serotonin transporter (5-HTT) protein, which has the job to control the release of serotonin (5-HT) from the synaptic terminals remove that from the synaptic cleft (39–42). Due to the importance of serotonin in impacting OCD, it is understandable that SLC6A4 might be the potential bio-marker for OCD treatment (6, 14, 43). Except for the polymorphism of 5-HTTLPR (SLC6A4) that previous research had already done with (33), the C alleles of rs16965628 (SLC6A4) have also been suggested to potentially lead to alterations in the serotonergic system (44). Therefore, rs16965628 is suitable for investigation in the present study, as it could be considered as a highly possible candidate gene foe OCD (40).

Moreover, GRIN2B seem also deserve the investigation as a possible candidate gene for OCD treatment, due to the previous finding of the association between G allele and OCD (45). Previous studies suggested that there is a significant correlation between the rs1019385 polymorphism of the N-methyl-D-aspartate 2B glutamate receptor (GRIN2B) and reduced glutamatergic concentration (Glx) in the anterior cingulate cortex (ACC); Individuals with the GG genotype demonstrated reduced Glx compared to those carrying the T allele (45, 46). As the reduced Glx was suggested to be related with OCD (47), rs1019385 (GRIN2B) could also be a suitable gene for investigation in the present study.

Furthermore, as another possible candidate gene, Catechol-O-methyltransferase (COMT) serves as a pivotal enzyme in the metabolic deactivation of dopamine and norepinephrine catecholamines, facilitating the catabolism of dopamine (DA) (48, 49). The Met (A) allele of Val158Met (G to A), commonly referred to as RS4680 (COMT), is proposed to decrease enzyme activity, elevate cortical dopamine signaling, and potentially play a role in OCD development (50, 51). Hence, RS4680 (COMT) emerges as another pertinent gene for investigation in the current study.

Given the limited amount of research investigating RS4680 (COMT), RS16965628 (SLC6A4), and RS1019385 (GRIN2B) as candidate genes for OCD treatment and their strong correlation with OCD, the present study aimed to investigate whether the polymorphism of those three genes would influence the therapeutic effect of rTMS and SSRIs in individuals with OCD. To achieve the aim, a study with an experimental, between-subject, double-blind design was conducted, with the recruitment of 36 untreated outpatients diagnosed with OCD.

## Methods and materials

### Participants

**Figure 1:**
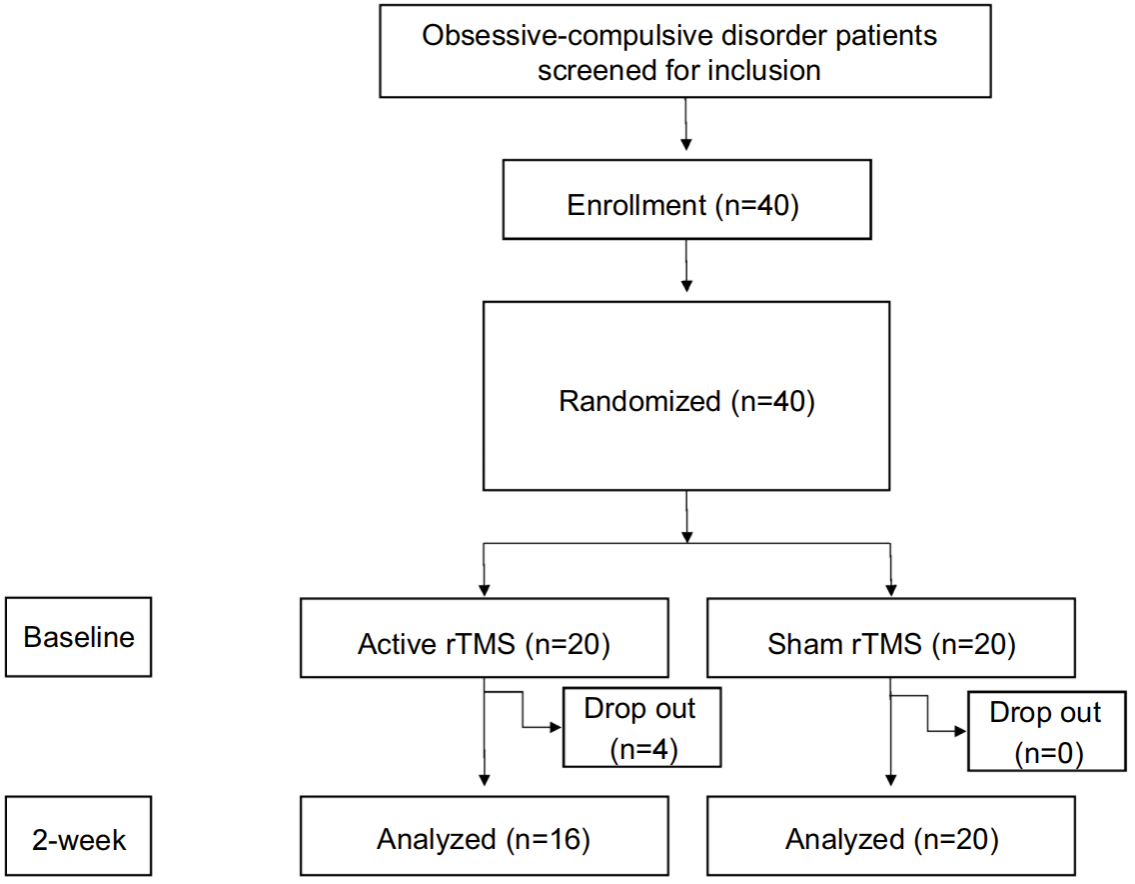
CONSORT diagram for the present experiment.

This study employed an experimental, between-subject, double-blind design. The participants consisted of 36 untreated outpatients diagnosed with OCD according to the Diagnostic and Statistical Manual of Mental Disorders 4th edition (DSM-IV) criteria (**Figure 1**). The mean age of the participants was 31.61, with 17 males (47.22%) and 19 females (52.78%). The study was granted ethical approval by the Ethics Committee of Wuxi Mental Health Center, China (clinical trials registry number: [ChiCTR1900023641]). Each participant has received informed consent before participating.

### Genotyping & therapy

All participants were randomly assigned to two different groups for an 8-week therapy. However, as the drop-out rate was extremely high after the second week, this study only analyzed the data of the first two weeks. Peripheral blood samples (5 mL) were collected from each participant using ethylenediaminetetraacetic acid (EDTA) vacuum tubes for genotyping purposes, specifically examining the polymorphism of RS4680 (COMT), RS16965628 (SLC6A4), and RS1019385 (GRIN2B). In the active rTMS group, participants received 1-Hz rTMS over the pre-supplementary motor area (pre-SMA) once per day, 5 days per week, for 2 weeks. The pre-SMA was identified as being 15% of the distance from the inion to the nasion along the anterior plane to the Cz (vertex) on the sagittal midline. For the sham rTMS group, the Neurosoft sham coil was positioned at the same location. Both groups received selective serotonin reuptake inhibitors (SSRIs) as part of their drug therapy. Further details regarding the genotyping and therapy used in this study can be found elsewhere (33).

### Assessments

Clinical evaluations were conducted at baseline and 2 weeks following the treatment timeline using three measuring tools: the Chinese version of Yale–Brown obsessive-compulsive scale (Y-BOCS), the Hamilton Anxiety Rating Scale (HAMA), and the 17-item Hamilton Depression Rating Scale (HAMD). The Chinese version of Y-BOCS was found to be a dependable computerized cognitive test, noted for its repeatability, sensitivity, and strong validity and reliability (33). There were also previous research affirmed the validity and sensitivity of the Chinese editions of HAMD and HAMA, underscoring their suitability for gauging clinical severity in patients and endorsing their ongoing utilization in research settings (52).

### Statistical analysis

Statistical analysis was performed using SPSS, employing the chi-square test, Related-Samples Friedman’s Two-Way Analysis of Variance, and the Scheirer-Ray-Hare Test. The Chi-square test was used to test the categories data such as gender. The Mann–Whitney test, Related-Samples Friedman’s Two-Way Analysis, and the Scheirer-Ray-Hare Test were used to test non-normally distributed data.

## Results

### Demographic characteristics of enrolled subjects

**Table 1** presents descriptive and statistical comparisons of the active rTMS and sham rTMS groups. At baseline, there were no significant differences between the active and sham groups in terms of demographics or baseline clinical ratings (**Table 1**).

**Table 1:** Demographic and clinical data of the OCD patients at baseline.

	Active rTMS group (n=16)	Sham rTMS group (n=20)	Statistics	p-value	Effect Size
Gender ª (male/female)	9/7	8/12	0.942	0.503	0.162
Age (years)	33.00	30.50	165.50	0.863	0.029
Y-BOCS	15.00	17.50	177.00	0.604	0.091
HAMA	12.00	10.50	154.00	0.863	0.032
HAMD	14.00	14.00	131.00	0.369	0.154

ªFor categories data(gender), Chi-square statistics are reported. Cramer’s V is used to estimate the effect size. For the age, Y-BOCS score, HAMA score, and HAMD score, the U statistic of Mann Whitney test was shown.

### Assessment of Y-BOCS, HAMA, and HAMD

**Figure 2:**
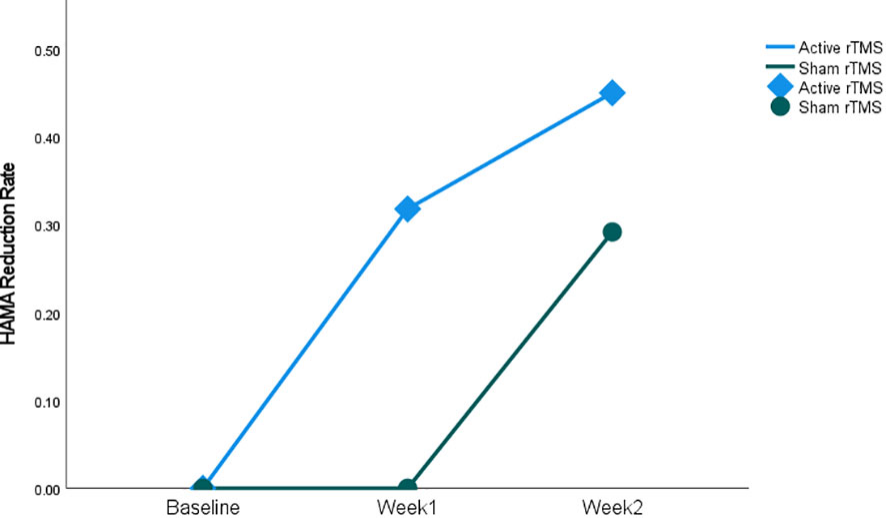
Change in HAMA score’s reduction rate in patients with OCD during the study. Data are shown at the time of inclusion in the study (baseline) and after the period of active or sham stimulation (weeks 1 and 2). *The active rTMS group’s week 1 reduction rate is significantly larger compared with the sham rTMS group (p < 0.05).

The result shows that both SSRIs and rTMS augmentation of SSRIs led to significant improvements in assessment scores. The Y-BOCS scores demonstrated a significant decrease over time. The Scheirer-Ray-Hare test analysis results (**Table 2**) for the two-week Y-BOCS assessment showed that the main effect of the therapy type was not significant (H = 2.91, p > 0.05), while the main effect of the therapy duration was significant (H = 12.19, p < 0.05, η2 = 0.12). Friedman test (**Table 3**) suggests that the assessment score had a significant change after therapy in both the active rTMS group (χ2 (2) = 17.44, p < 0.01, W = 0.55) and sham rTMS group (χ2 (2) = 9.58, p < 0.01, W = 0.24). *Post hoc* analysis with Wilcoxon signed-rank tests was conducted with a Bonferroni correction applied, resulting in a significance level set at p < 0.017. In the active rTMS group, Y-BOCS scores significantly decreased from baseline to week 1 (Z = -2.96, p < 0.017) and from baseline to week 2 (Z = -3.33, p < 0.017), while the decrease from week 1 to week 2 was not significant (Z = -1.74, p = 0.082). Similarly, in the sham rTMS group, Y-BOCS scores significantly decreased from baseline to week 2 (Z = -2.45, p<0.017), while the decrease from baseline to week 1 (Z = -2.12, p = 0.034) and from week 1 to week 2 (Z = -1.55, p = 0.120) was not significant (**Table 3**). HAMA scores exhibited significant changes after therapy in both the active group (χ2 (2) = 7.77, p < 0.05, W = 0.24) and sham group(χ2 (2) = 11.40, p < 0.01, W = 0.29) (**Table 3**). Besides, in the active rTMS group, a significant change in assessment scores was found both from baseline to week 1 (Z = -2.81, p < 0.017) and from baseline to week 2 (Z = -2.88, p < 0.017). In the sham rTMS groups, a significant change was only found between baseline and week 2 (Z = -2.66, p < 0.017) (**Table 3**). Additionally, the analysis of the two-week HAMD assessment showed that the main effect of the therapy duration was significant (H = 10.50, p < 0.05, η2 = 0.10) (**Table 2**). Patients in the active rTMS group demonstrated a significantly larger reduction rate at week 1 compared to the patients in the sham group (U = 93.50, p < 0.05, r = 0.354) (**Figure 2**). The HAMD scores showed a significant decrease over time in both the active rTMS (χ2 (2) = 16.00, p < 0.05, W = 0.50) and sham rTMS groups (χ2 (2) = 11.86, p < 0.05, W = 0.30) (**Table 3**).

**Table 2:** Scheirer-ray-hare test of the assessment scores.

		Df	Sum Sq	H	p.value	Eta Squared(η2)
**Y-BOCS**	**rTMS(R)**	1	2846	2.91	0.09	0.031
	**Time(T)**	2	11906	12.19	**0.00**	0.117
	R*G	2	274	0.28	0.87	0.003
	**Residuals**	102	89519			
**HAMA**	**rTMS(R)**	1	595	0.61	0.43	0.006
	**Time(T)**	2	4718	4.83	0.09	0.046
	R*G	2	688	0.70	0.70	0.007
	**Residuals**	102	98519			
**HAMD**	**rTMS(R)**	1	545	0.56	0.46	0.006
	**Time(T)**	2	10274	10.50	**0.01**	0.100
	R*G	2	1213	1.24	0.54	0.013
	**Residuals**	102	92638			

The Scheirer-Ray-Hare Test was used to analyze the influence of the treatment type on the relationship between the therapy duration and the assessment scores. Eta square is calculated to estimate the effect size (Small Effect: 0.01- 0.06; Medium effect: 0.06 - 0.14; Large Effect ≥ 0.14). The bold values are statistically significant.

**Table 3:** Assessment scores of OCD patients after treatment.

	Group	Baseline	Week1	Week2	Df	Test Statistic(χ2)	Effect Size
**Y-BOCS**	Active	15	12.5*	10.5**	2	17.44**	0.545
	Sham	17.5	15	12**	2	9.58**	0.239
**HAMA**	Active	12	7.5**	5**	2	7.77*	0.243
	Sham	10.5	8.5	7.5**	2	11.40**	0.285
**HAMD**	Active	14	12.5*	5**	2	16.00**	0.500
	Sham	14	9*	8**	2	11.86**	0.296

**p<0.01 compared with the baseline *p<0.05 compared with the baseline. Assessment scores are presented as median. The Related-Samples Friedman’s Two-Way Analysis of Variance by Ranks was used to analyze the effect of the treatment on the assessment scores over time in different groups. Kendall’s W was calculated to estimate the effect size. The bold values are statistically significant.

### Effects of genotype on RS4680, RS16965628 and RS1019385

**Figure 3:**
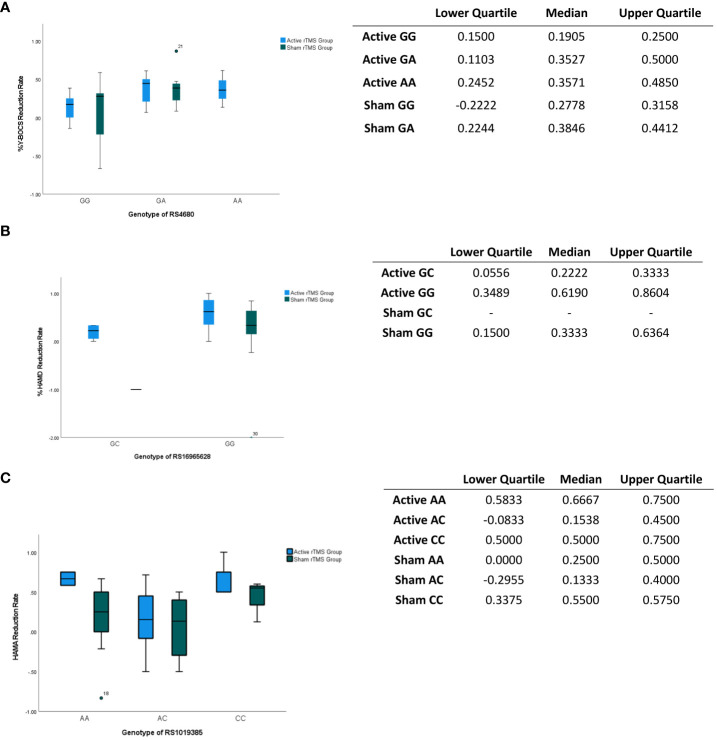
Impacts of gene locus on the assessments score. **(A)** *Impacts of gene locus RS4680 on the Y-BOCS reduction at week 2.* The gene locus RS4680 exerts a substantial impact on the Y-BOCS score, as evidenced by its statistical significance (p<0.05). The table shows the interquartile and median of the boxplot. **(B)** *Impacts of gene locus RS16965628 on the HAMD reduction at week 2.* The gene locus RS12965628 exerts a notable influence on the reduction of the HAMD score, as evidenced by its statistically significant effect (p<0.05). The table shows the interquartile and median of the boxplot. **(C)** *Impacts of gene locus RS1019385 on the HAMA reduction at week 2.* The gene locus RS1019385 exerts a notable influence on the reduction of the HAMD score, as evidenced by its statistically significant effect (p<0.05). The table shows the interquartile and median of the boxplot. The point shows the outlier.

The first significant difference was observed in the RS4680 genotype. No significant differences were found in general baseline characteristics between different genotypes. However, significant genotype-related effects on the intervention outcomes were identified (**Table 4**). Patients with the GG genotype showed a smaller improvement in Y-BOCS scores (H = 6.374, p < 0.05, η2 = 0.19); and greater variability in Y-BOCS score was found in Sham rTMS groups of GG genotype than in others. (**Figure 3A**). Additionally, an interaction between rTMS intervention and RS4680 genotype was found to have effects on HAMA (H = 4.645, p < 0.05, η2 = 0.14) and HAMD (H = 5.549, p < 0.05, η2 = 0.17) scores at week 2, suggesting that genotypes may influence the outcome of rTMS. Regarding the RS16965628 genotype, the analysis results (**Table 2**) showed that the main effect of rTMS (H = 4.514, p < 0.05, η2 = 0.15) was significant for the HAMD Week2 Reduction Rate. The rTMS intervention significantly improved HAMD scores to a greater extent in the active group, regardless of whether patients had the GC or GG genotype, compared to the sham group; and the improvement of the HAMD score of patients carrying the GC genotype are more concentrated than the improvement of the patients with GG genotype (**Figure 3B**). Furthermore, the RS16965628 genotype exerted a significant influence on the reduction of HAMD scores (H = 6.361, p < 0.05, η2 = 0.20), with patients carrying the GG genotype showing greater improvement compared to C allele carriers (**Table 4** and **Figure 3B**). However, the interaction between intervention type and genotype did not impact the rate of HAMD score reduction (H = 0.024, p > 0.05) (**Table 4**). For RS1019385, polymorphism of the gene also demonstrated a significant influence on HAMA score reduction (H = 6.057, p < 0.05, η2 = 0.19). Specifically, individuals with the CC genotype exhibited greater improvement in HAMA scores compared to those with other genotypes; and the reduction rate of the HAMA score are found to be more volatility in AC genotype carrier (**Figure 3C**).

**Table 4:** Impacts of gene RS4680, gene RS16965628, and RS1019385 on week2.

			Df	Sum Sq	H	p.value	Eta Squared(η2)
**RS4680**	**HAMA Week2 Score**	**rTMS(R)**	1	156.74	1.421	0.23	0.048
		**Genotype(G)**	2	165.40	1.500	0.47	0.051
		R*G	1	512.30	4.645	**0.03**	0.142
		**Residuals**	31	3087.68			
	**HAMD Week2 Score**	**rTMS(R)**	1	121.89	1.106	0.29	0.038
		**Genotype(G)**	2	116.13	1.054	0.59	0.037
		R*G	1	611.44	5.549	**0.02**	0.167
		**Residuals**	31	3059.12			
	**Y-BOCS Week2 Reduction Rate**	**rTMS(R)**	1	0.28	0.003	0.96	0.000
		**Genotype(G)**	2	707.22	6.374	**0.04**	0.185
		R*G	1	3.76	0.034	0.85	0.001
		**Residuals**	31	3120.51			
**RS16965628**	**HAMD Week2 Reduction Rate**	**rTMS(R)**	1	472.78	4.514	**0.03**	0.151
		**Genotype(G)**	1	666.25	6.361	**0.01**	0.200
		R*G	1	2.53	0.024	0.87	0.000
		**Residuals**	31	2664.23			
**RS1019385**	**HAMA Week2 Reduction Rate**	**rTMS(R)**	1	265.00	2.533	0.11	0.092
		**Genotype(G)**	2	633.78	6.057	**0.05**	0.194
		R*G	2	159.08	1.520	0.46758	0.057
		**Residuals**	29	2627.60			

The Scheirer-Ray-Hare Test was used to analyze the influence of the genotype on the relationship between the therapy type and the assessment scores. The eta square is calculated to estimate the effect size (Small Effect: 0.01- 0.06; Medium effect: 0.06 - 0.14; Large Effect ≥ 0.14). The bold values are statistically significant.

## Discussion

The present study provides evidence that both SSRIs and rTMS augmentation of SSRIs led to significant improvements in OCD symptoms following a two-week intervention. Additionally, polymorphisms of RS4680, RS16965628, and RS1019385, were found to be associated with treatment response. The study reveals that individuals with the GG allele of RS4680 (*COMT*) exhibited a relatively poorer response in terms of reducing Y-BOCS scores, not only to SSRIs treatment but also to combined treatment involving SSRIs and rTMS. This finding may be attributed to the decreased susceptibility of patients with the GG allele (RS4680) to rTMS or the combination treatment. RS4680, also known as Val158Met (G to A), is closely linked to the risk of OCD (50, 51). It has been suggested that the Met (A) allele reduces the enzyme activity, enhances cortical dopamine signaling, and contributes to OCD. This explanation aligns with the current study’s finding that patients with the GG allele of RS4680 lack the A (Met) allele and are therefore less susceptible to both OCD and its treatments (50).

Regarding the serotonin transporter protein gene (*SLC6A4*), no significant association was observed between different genotypes and Y-BOCS scores. However, the C allele at RS16965628 (*SLC6A4*) predicted improvements in HAMD scores, indicating that patients with the C allele may be more responsive to rTMS augmentation therapy of SSRIs in reducing depressive symptoms. This result could be said to keep consistent with previous experimental findings on the association between the C allele and OCD patients (35, 44), highlighting the strong connection between serotonin and the depressive symptoms of OCD patients. The potential bio-marker of the C allele in RS16965628 in *SLC6A4* further supports the notion proposed by Zhang (33) in 2019 that *SLC6A4* could serve as a candidate gene for the combined treatment of SSRI and rTMS.

Furthermore, the study results indicate that GG allele in RS1019385 significantly benefits in reducing HAMA scores, implying that patients with this specific genotype may be more susceptible to rTMS augmentation therapy of SSRIs in alleviating anxiety symptoms. This finding might be seen as be supported by Arnold (45) who established a significant association between ACC Glx levels and the *GRIN2B*-RS1019385 polymorphism, with individuals carrying the GG genotype showing reduced Glx compared to T allele carriers. The G allele of RS1019385 represents a variant in the promoter region, potentially leading to decreased transcription and impacting glutamatergic neurotransmission, thereby possibly contributing to OCD (45–47).

There are several limitations of this study, including a small sample size, high drop-out rate and a short trial period of two weeks. These may account for the low effect size of some of the results. These limitations might have affected the reliability and validity of the current study. Therefore, future research should include a larger and more diverse sample size and conduct longer interventions, some better welfare might be provided to minimize withdrawal rates.

In conclusion, the A allele of *COMT* RS4680, the C allele of *SLC6A4* RS16965628, and the GG allele of *GRIN2B* RS1019385 may serve as bio-markers for the combined treatment of rTMS and SSRIs in OCD, influencing Y-BOCS, HAMD, and HAMA scores, respectively. Due to limitations in sample size and trial duration, further research is needed to validate these findings. The findings of this study provide valuable insights for further investigation and the implementation of precision treatment for OCD.

## Data availability statement

The original contributions presented in the study are included in the article/supplementary materials, further inquiries can be directed to the corresponding author/s.

## Ethics statement

The studies involving humans were approved by Ethics Committee of Wuxi Mental Health Center, China (clinical trials registry number: [ChiCTR1900023641]). The studies were conducted in accordance with the local legislation and institutional requirements. The participants provided their written informed consent to participate in this study.

## Author contributions

LC: Conceptualization, Methodology, Software, Validation, Visualization, Writing – original draft, Writing – review & editing. YW: Conceptualization, Methodology, Software, Validation, Visualization, Writing – original draft, Writing – review & editing. JY: Data curation, Methodology, Resources, Writing – review & editing. KZ: Data curation, Methodology, Resources, Writing – review & editing. YZ: Supervision, Validation, Writing – review & editing. XF: Conceptualization, Data curation, Formal analysis, Investigation, Project administration, Supervision, Validation, Writing – review & editing. GW: Project administration, Supervision, Writing – review & editing.
